# Theranostics in Oncology—Thriving, Now More than Ever

**DOI:** 10.3390/diagnostics11050805

**Published:** 2021-04-29

**Authors:** Rudolf A. Werner, Takahiro Higuchi, Martin G. Pomper, Steven P. Rowe

**Affiliations:** 1 Department of Nuclear Medicine and Comprehensive Heart Failure Center, University Hospital Würzburg, 97080 Würzburg, Germany; thiguchi@me.com; 2 Okayama University Graduate School of Medicine, Dentistry and Pharmaceutical Sciences, Okayama 700-8558, Japan; 3 The Russell H. Morgan Department of Radiology and Radiological Science, Johns Hopkins University School of Medicine, Baltimore, MD 21287, USA; mpomper@jhmi.edu (M.G.P.); srowe8@jhmi.edu (S.P.R.); 4 The James Buchanan Brady Urological Institute and Department of Urology, Johns Hopkins University School of Medicine, Baltimore, MD 21287, USA

**Keywords:** theranostics, somatostatin receptor (SSTR), prostate-specific membrane antigen (PSMA), prostate cancer, neuroendocrine neoplasms (NEN), neuroendocrine tumors (NET), meningioma, norepinephrine transporter, neuroblastoma

## Abstract

Tracing its roots back to the 1940s, theranostics in nuclear oncology has proved successful mainly due to the beneficial effects of image-guided therapeutic concepts for patients afflicted with a variety of different cancers. The majority of these treatments are not only characterized by substantial prolongation of progression-free and overall survival, but are also generally safe, rendering theranostic agents as an attractive treatment option in various clinical scenarios in oncology. In this Special Issue *Novel Theranostic Agents*, nine original articles from around the globe provide further evidence on the use of the theranostic concept for neuroendocrine neoplasm (NEN), prostate cancer (PC), meningioma, and neuroblastoma. The investigated diagnostic and therapeutic radiotracers target not only established structures, such as somatostatin receptor, prostate-specific membrane antigen or norepinephrine transporter, but also recently emerging targets such as the C-X-C motif chemokine receptor 4. Moreover, the presented original articles also combine the concept of theranostics with in-depth read-out techniques such as radiomics or novel reconstruction algorithms on pretherapeutic scans, e.g., for outcome prediction. Even 80 years after its initial clinical introduction, theranostics in oncology continues to thrive, now more than ever.

Tracing its roots back to the 1940s [1], theranostics in nuclear oncology has seen an unprecedented success, mainly due the beneficial effects of such image-guided therapeutic concepts for patients afflicted with radioiodine avid thyroid cancer [2]. Recent years, however, have witnessed an expanded use of novel molecular imaging agents tied to an individually tailored treatment decision [3,4]. Such a theranostic approach enables systemic or locoregional radiation of various cancer entities with mainly β-emitting radionuclides, which are linked to the identical molecule used for positron emission tomography (PET) or single-photon emission computed tomography (SPECT) imaging [4]. The more widespread adoption of theranostics is further fueled by the encouraging results of recently published major trials like NETTER-1 for imaging and therapy of neuroendocrine neoplasms (NEN) targeting the somatostatin receptor (SSTR) [5] or reports on the beneficial effects of this concept for the treatment of prostate cancer (PC), as evidenced by the prospective LuPSMA or TheraP trials [6,7]. In this Special Issue, nine original articles from all around the globe provide further evidence on the use of the theranostic concept for NEN, PC, meningioma, and neuroblastoma, targeting various proteins on the tumor cell surface, including SSTR, prostate-specific membrane antigen (PSMA), norepinephrine transporter (NET) and C–X–C motif chemokine receptor 4 (CXCR4).

First, Wakabayashi et al. report on the use of post-therapeutic [^131^I]*meta*-iodobenzylguadinine ([^131^I]mIBG) scans in children afflicted with neuroblastoma, elegantly demonstrating that post-therapy imaging can provide further information on the extent of disease relative to pretherapeutic SPECT performed for diagnostic purposes. As such, [^131^I]MIBG scans acquired after therapy should be closely examined so that relevant sites of disease will not be missed [8].

For NEN, SSTR-directed theranostics is considered the standard-of-care at a large number of theranostics centers [9]. Ohlendorf and coworkers investigated whether markers of systemic inflammation, which are routinely assessed in the clinic, have early predictive and prognostic value for patients with advanced gastro-entero-pancreatic (GEP) NEN scheduled for SSTR-targeted endoradiotherapy. C-reactive protein emerged as a helpful tool to differentiate between low- vs. high-risk individuals prone to treatment failure during follow-up, thereby suggesting that tumor-driven systemic inflammatory networks are of importance for treatment response or prognosis [10].

For GEP NEN patients, the mTOR inhibitor everolimus is endorsed by current guidelines [11]. In a bi-centric study, Wetz and coworkers therefore performed an in-depth analysis of mathematically extracted radiomic feature metrics derived from pre-therapeutic [^111^In]DTPA-octreotide scintigraphy in GEP-NEN patients treated with everolimus. They identified the radiomics parameter of lesional asphericity (ASP) as an independent predictor for outcome, thereby suggesting that ASP may serve as a risk stratification tool prior to treatment onset [12]. Nonetheless, therapeutic options, in particular in high-grade GEP-NEN, are intensively sought [13].

Weich et al. performed a thorough evaluation of the novel CXCR4-targeting PET compound [^68^Ga]Pentixafor, which can also be used in a theranostic setting using its ^177^Lu-labeled twin Pentixather, in hematological malignancies [14,15]. In a molecular binding assay study, modulation of the Wnt pathway enhanced CXCR4 expression in established and novel NEN cell lines, along with increased accumulation of [^68^Ga]Pentixafor [16]. In another study enrolling GEP-NEN patients, the latter compound did not provide superior information relative to the current standard radiotracer 2-deosy-2-[^18^F]fluoro-D-glucose ([^18^F]FDG) in this clinical scenario. Nonetheless, uptake was still substantial on CXCR4-directed imaging and the lesion-based heterogeneity of [^18^F]FDG-avid and [^68^Ga]Pentixafor-negative lesions, and vice versa, should be further explored for outcome prediction [17].

However, the concept of SSTR theranostics is not only used for GEP-NEN, but has also been expanded toward meningiomas and to select individuals who would most likely benefit from treatment. Pre-therapeutic [^68^Ga][DOTA0-Phe1-Tyr3] octreotide ([^68^Ga]DOTATOC) PET is highly useful in this scenario [18]. In this regard, Graef et al. investigated different image time-points after injection in meningioma patients and reported a 100% lesion detection rate in all investigated subjects by 10 min. p.i. Such information may be helpful for scheduling patients in a busy PET practice, e.g., by allowing for substantially shortened uptake times [19].

Last, this Special Issue also deals with PSMA-directed imaging and treatment for PC. As with any imaging modality, indeterminate findings may occur, which should not provide misleading information to the referring urologists [20]. Therefore, standardized frameworks for PSMA scan interpretation have been introduced, including PROMISE, E-PSMA or the PSMA-Reporting and Data System (PSMA-RADS) [21,22,23]. The latter framework includes PSMA-RADS-3A lesions, which describes findings that have equivocal uptake in a soft tissue site that would be typical for metastatic PC, such as pelvic or retroperitoneal lymph nodes. The rather low radiotracer uptake, however, renders such lesions as indeterminate, requiring further work-up. This is in contrast to PSMA-RADS-4 lesions, which are characterized by an intense radiotracer uptake in sites typical for PC and therefore, have a high likelihood of being malignant [23]. Khatri et al. investigated state-of-the-art reconstruction algorithms for the PSMA-targeted compound [^18^F]DCFPyL and reported on the use of point-spread function (PSF) for a more appropriate categorization of indeterminate PSMA-RADS-3A to (more definitive) PSMA-RADS-4 lesions, which renders PSF reconstructions for PSMA-targeted PET as a useful tool to increase interpretative certainty [24].

The last two original articles refer to the use of [^177^Lu]PSMA-617 for radioligand therapy for PC. First, Völter et al. analyzed PC patients who had undergone PSMA-targeted therapy [25]. For PSMA theranostics, post-therapeutic dosimetry may be a useful tool to tailor the treatment protocol to a patient’s individual needs, e.g., by providing absorbed doses to organs at risk or the tumor. Nonetheless, such a whole-body tumor dosimetry applied to all relevant sites of disease is challenging [26]. Therefore, a single index-lesion-based post-therapeutic SPECT dosimetry was performed on scans that have been acquired after injection of [^177^Lu]PSMA-617 and Völter and colleagues demonstrated that this approach provided a fast and feasible dosimetry read-out for response assessment. Therefore, relative to time-consuming dosimetry of the entire tumor burden, such a simplified procedure of analyzing post-therapeutic SPECT may be more practical for clinical routine [25]. Post-therapeutic SPECT, however, is not available prior to treatment and reliable outcome predictors before commencing PSMA-directed treatment would be desirable. As such, Moazemi et al. investigated radiomic features from pretherapeutic [^68^Ga]PSMA PETs and reported on the usefulness of such mathematically extracted scan signatures for outcome prediction in patients scheduled for [^177^Lu]PSMA [27].

We hope that the readers of this Special Issue enjoy the broad content as much as we did. Taken together, even 80 years after the initial introduction of using radioiodine for thyroid cancer, theranostics in oncology is definitively alive and increasingly relevant.

## Data Availability

Not applicable.
